# MODY5 and 17q12 Microdeletion Syndrome: Phenotype Variability, Prenatal and Postnatal Counseling

**DOI:** 10.3390/genes16091002

**Published:** 2025-08-25

**Authors:** Paolo Fontana, Claudia Costabile, Mariateresa Falco, Maria Rosaria Barillari, Fortunato Lonardo

**Affiliations:** 1 Medical Genetics Unit, P.O. Gaetano Rummo, A.O.R.N. San Pio, 82100 Benevento, Italy; mariateresa.falco@aornsanpio.it (M.F.); fortunato.lonardo@gmail.com (F.L.); 2 AOU “L. Vanvitelli”, Dipartimento di Salute Mentale e Fisica e Medicina preventiva, Servizio di Audiologia e Foniatria, 80138 Napoli, Italy; claudia.costabile@unicampania.it (C.C.);

**Keywords:** *HNF1B*, 17q12 deletion, MODY5, polycystic kidney, prenatal diagnosis, neurodevelopmental disorders

## Abstract

Maturity-Onset Diabetes of the Young Type 5 (MODY5) is caused by heterozygous pathogenic variants in the *HNF1B* gene, encoding the transcription factor hepatocyte nuclear factor-1β. *HNF1B* haploinsufficiency typically leads to young-onset non-immune diabetes and highly variable renal involvement, whose more frequent features are bilateral kidney cysts and renal hypodysplasia. Kidney cysts or echogenic kidneys can be identified by ultrasonography in the prenatal period, but the renal involvement can also start in childhood or later. Notably, a recurrent microdeletion syndrome at 17q12 (deleting *HNF1B* plus ~15 neighboring genes) accounts for ~40–50% of cases. The 17q12 deletion is a contiguous gene syndrome and affected individuals present with a complex phenotype, including neurodevelopmental disorders, liver and pancreas abnormalities, and other congenital defects. When counseling the patient and the parents, the clinician must consider multiple factors, including the molecular defect and the age of onset of the symptoms, with particular attention to prenatal diagnosis. A multidisciplinary approach and an early diagnosis are essential for the management of these conditions.

## 1. Renal Cysts and Diabetes Syndrome: One of the Inherited Forms of Diabetes

Maturity-Onset Diabetes of the Young Type 5 (MODY5), also referred to as Renal Cysts and Diabetes (RCAD) syndrome, is specifically attributed to heterozygous variants or deletions within the hepatocyte nuclear factor 1-beta (*HNF1B*) gene, also known as *TCF2*. This particular subtype constitutes approximately 2% to 6% of all diagnosed MODY cases [1].

Maturity-Onset Diabetes of the Young (MODY) is a distinct, rare form of monogenic diabetes mellitus, characterized by its autosomal dominant inheritance pattern and an early age of onset, typically before 25 years [2]. This group of conditions primarily stems from impaired insulin production or release by pancreatic beta cells. The foundational concept of MODY was introduced by Robert Tattersall and Stefan Fajans in their pivotal 1975 publication in the journal *Diabetes*. Their work described a cohort of young individuals presenting with non-insulin-dependent diabetes, a presentation that diverged from the then-understood categories of juvenile-onset (Type 1) and adult-onset (Type 2) diabetes. This initial characterization was instrumental in distinguishing MODY as a unique entity within the broader spectrum of diabetes.

Despite its distinct genetic basis, MODY accounts for an estimated 1% to 5% of all diabetes diagnoses. A significant challenge in clinical practice has been the high rate of misdiagnosis, with 50% to 90% of MODY cases initially classified as Type 1 or Type 2 diabetes due to overlapping clinical features. This diagnostic ambiguity highlighted an early limitation in diabetes classification, which relied heavily on phenotypic observation. The subsequent identification of specific genetic causes for MODY subtypes, including MODY5, marked a crucial advancement in diagnostic precision. This evolution from a purely clinical assessment to genotypic confirmation has profound implications for patient management and family screening, emphasizing the growing role of molecular diagnostics in contemporary medicine. To date, over 20 genes have been identified as causative for various MODY subtypes, all playing roles in pancreatic beta-cell function, with *GCK*, *HNF1A*, *HNF4A*, and *HNF1B* being among the most frequently implicated. The pioneering clinical observations by Tattersall and Fajans effectively established the concept of hereditary diabetes in younger, non-obese individuals, thereby laying the groundwork for subsequent investigations into monogenic causes of diabetes and paving the way for the later discovery of specific gene mutations like *HNF1B*.

## 2. The Major MODY5 Associated Gene: HNF1B

*HNF1B* was identified as the causative gene for MODY5 in 1997, appearing in *Nature Genetics*. This pivotal study provided the first molecular basis for this distinct form of monogenic diabetes [3].

**Figure 1:**
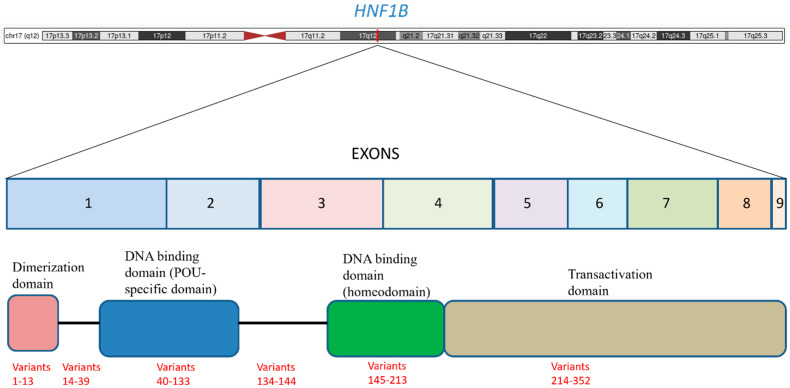
*HNF1B* is located on the long arm of chromosome 17 (17q12) and it consists of nine exons. The figure shows the main functional domains and the distribution of the variants. The variants are listed according to the schema proposed in Table 1 (for example, the variants from 1 to 13 of Table 1 are within the dimerization domain).

The hepatocyte nuclear factor 1 beta (*HNF1B*) gene, located on chromosome 17q12, consists of nine exons and is a POU (Pit-Oct-Unc) homeodomain transcription factor belonging to the homeodomain-containing superfamily. Its protein product, HNF-1β, is characterized by three key functional domains: a dimerization domain, a DNA-binding homeodomain, and a transactivation domain (see Figure 1). The dimerization domain allows the protein to form either homodimers with itself or heterodimers with its paralog, *HNF1A*, which is essential for its regulatory function. The DNA-binding homeodomain enables the protein to bind to specific DNA sequences, thereby modulating the expression of numerous target genes. This domain is actually bipartite, consisting of an N-terminal POU-specific domain (POU_S) and a C-terminal homeodomain (POU_H), which together recognize and bind to the specific DNA sequence 5′-GTTAATNATTAAC-3′ in the promoter regions of target genes. The transactivation domain recruits coactivators and co-repressors to modulate gene expression [4]. This pleiotropic gene is widely expressed in various epithelial tissues, with high levels found in the kidneys, pancreas, liver, lungs, and the urogenital and intestinal tracts. The primary functions of *HNF1B* are deeply rooted in embryonic development, where it plays a pivotal role in the morphogenesis of organs like the kidneys, pancreas, liver, and genitourinary tract. It is also a key regulator of cellular processes such as the cell cycle, apoptosis, and the maintenance of specialized epithelia. The widespread expression of *HNF1B* during embryogenesis directly accounts for the multisystemic clinical manifestations observed in individuals with MODY5. However, the functions of *HNF1B* extend beyond simply regulating organogenesis; for example, in renal physiology, HNF-1β controls a transcriptional network that maintains tight junction integrity and cell structure in the distal nephron. It also directly regulates genes involved in ion transport and primary cilia formation, such as *PKD2*, *PKHD1*, and *UMOD*, disruptions of which are implicated in cyst formation and kidney disease. In the pancreas, HNF-1β regulates genes essential for beta-cell function and insulin production, directly linking its haploinsufficiency to the onset of diabetes [5,6]. The discovery of the disease-causing gene allowed the comprehension of the underlying developmental pathology, providing the link between the genetic defect and the diverse, often non-diabetic, symptoms exhibited by patients. This understanding is fundamental to conceptualizing *HNF1B*-associated disease as a broader condition, not merely a form of diabetes.

Inheritance is autosomal dominant, but up to 30–60% of cases are de novo, so a family history may be absent [4,7]. Reported variants include point mutations (missense, frameshift, nonsense, splice site) and whole-gene deletions [8]. Single nucleotide variants cluster in exons 2–4 (homeodomain) and intron 2 splice sites. A recurrent microdeletion syndrome at 17q12 (deleting *HNF1B* plus ~15 neighboring genes) accounts for ~40–50% of cases, as discussed in a later section. Among non-deletion cases, ~52% of alleles are missense, ~29% frameshift, ~15% nonsense, and the remainder are splicing variants, small deletions, insertions, or indels. The genotype shows no significant correlation with renal outcomes or other extrarenal manifestations [8,9]. A summary of the published variants is reported in Table 1.

## 3. 17q12 Deletions

The 17q12 deletion, encompassing, among the other genes, *HNF1B*, is a recurrent 1.4 Mb deletion, which usually involves the same sequence, with genomic coordinates (GRCh38): 36,458,167–37,854,616. The 17q12 region is considered an hot spot for copy number variants because it is flanked by segmental duplications on each side. These repetitive regions of DNA can misalign during meiosis, causing the deletions [10].

The 17q12 deletion removes 15 genes, among which, presumably, *HNF1B*, is not the only one to exhibit a phenotype caused by haploinsufficiency. The gene *LHX1*, involved in the recurrent deletion, plays a role in the development of the brain and it is supposed to contribute to the neurodevelopmental phenotypes [11], while *GGNBP2* is involved in the regulation of kidney development and the reproductive system [12].

The phenotype associated with the 17q12 deletion significantly differs from the phenotype associated with *HNF1B* single nucleotide variants. The neurobehavioral features, the ocular findings, and the male congenital genital defects are mainly associated with the deletions, as discussed in detail in the specific sections. Some authors suggest that intragenic *HNF1B* pathogenic variants could be related to a worse renal phenotype, but other authors did not detect a significant difference from the renal phenotype of those with 17q12 deletions.

## 4. Maturity-Onset Diabetes of the Young

MODY5 is defined by young-onset non-immune diabetes due to *HNF1B* haploinsufficiency.

**Age at onset** Most patients develop diabetes in adolescence or young adulthood (mean ~25 years), although onset can range from neonatal to late adulthood. Amaral et al. [4] reported a median diabetes diagnosis age of 28 years. Patients with 17q12 deletions may develop diabetes later than point mutation cases. As MODY5 is rare (<5% of MODY) and overlaps type 1/2 diabetes, it is often misdiagnosed. Many patients are initially diagnosed, and treated, as T1D or T2D.

**Course and insulin requirement** Progressively worsening β-cell dysfunction and a decline in insulin secretion leads to many patients requiring insulin. In one large series, ~49% were on insulin at diagnosis and 79% by follow-up [13]. Unlike *HNF1A*-MODY, MODY5 often shows a limited response to oral hypoglycemic agents. While some patients may initially respond to sulfonylureas, most will eventually require insulin for glycemic control [7,14]. Management can be complex because of the associated renal phenotype and other extra-pancreatic complications.

**Clinical presentation** Initial symptoms may include polyuria, weight loss, and fatigue. Ketoacidosis is uncommon but reported. Diabetes can be the initial manifestation of the syndrome or it may appear years after kidney disease. In patients with *HNF1B* variants, diabetes is the first feature occurring in 50% of the patients, while kidney disease occurs first in the other half of the cases. The disease course is more heterogeneous in patients with 17q12 deletions, who often start genetic evaluations after the diagnosis of a neurodevelopmental disorder [4,15].

**Laboratory findings** Diabetes is non-autoimmune: pancreatic autoantibodies (GAD, IA-2, ICA) are typically not detectable. C-peptide levels are inappropriately low (β-cell deficiency) even when glycemia is modest. Glycemic control deteriorates over years if untreated. Chronic microvascular complications (retinopathy, neuropathy) depend on the diabetes duration [16,17].

## 5. Renal Manifestations

*HNF1B* is critical for nephron and genitourinary development [18]. Renal disease is often the earliest or most prominent feature of *HNF1B*-MODY. Patients commonly have bilateral kidney cysts and/or hypodysplasia (the “renal cysts and diabetes” syndrome), but the spectrum is broad.

Key renal findings include:

1. **Cystic kidney disease**: Bilateral cortical and medullary cysts (sometimes resembling ADPKD) are frequent. Many patients show multiple kidney cysts on imaging, even if renal function is preserved [19].
2. **Renal hypodysplasia/agenesis**: Unilateral or bilateral small kidneys, hyperechogenic kidneys on ultrasound, or solitary kidney are common. Up to 30–40% have renal hypoplasia or agenesis [20].
3. **Tubulointerstitial kidney disease (ADTKD)**: Some patients develop slowly progressive cystic kidney disease with tubulointerstitial fibrosis. *HNF1B*-related ADTKD is now recognized as a common monogenic CKD cause [21].
4. **Congenital anomalies of the kidney/urinary tract (CAKUT)**: A range of structural anomalies can occur (horseshoe kidney, duplex collecting systems, hydronephrosis) [8].
5. **Nephrogenic diabetes insipidus**: Partial nephrogenic DI (polyuria and dilute urine) are occasionally described [8].
6. **Electrolyte disturbances**: Chronic renal magnesium wasting leads to hypomagnesemia in many patients. Hypokalemia is also reported. Hyperuricemia/gout can occur at a young age [22].
7. **End-stage renal disease (ESRD)**: Cystic kidney disease can lead to renal failure, often developing in adolescence/adulthood but not in childhood [23]. In one series, 8 of 10 patients had cystic kidney disease and several required transplant; kidney disease may precede or outpace the onset of the diabetes [4]. The progression to ESRD appears to be less in patients with 17q12 deletions compared to patients with *HNF1B* point variants [13].

## 6. Neurodevelopmental Disorders

Neurodevelopmental disorders (NDD) are one of the main features of 17q12 recurrent microdeletion. The effects on neurodevelopment of *HNF1B* intragenic single nucleotide variants (SNVs) are still a matter of debate, as some authors suggest that *HNF1B* loss-of-function can contribute to intellectual disability and other disorders, while other studies indicate that the prevalence of NDD in patients with *HNF1B* SNV does not differ significantly from the prevalence observed in the general population [24,25].

From a molecular standpoint, 17q12 deletion results in haploinsufficiency not only of *HNF1B* but also of other genes critical for neurogenesis. Notably, *LHX1*, which is involved in cortical neuronal migration, as well as *PIGW* and *PCGF2*, which are associated with glycosylation defects and epigenetic dysregulation, respectively, have been implicated in developmental delay and epilepsy [26,27]. Experimental studies in murine models suggest that *HNF1B* haploinsufficiency may interfere with hindbrain embryogenesis, while the combined deletion of adjacent genes may exert synergistic effects impairing synaptogenesis and neuronal plasticity [28].

In a large-scale systematic analysis of 695 individuals—416 with 17q12 microdeletion and 279 with intragenic *HNF1B* mutations—the overall prevalence of neurodevelopmental disorders (NDDs) was significantly higher (*p*  <  0.001, two-sided) in the microdeletion group (25.2%) compared to the intragenic mutation group (6.8%), suggesting a distinct contribution of the adjacent genes to the emergence of the neurocognitive phenotype [29]. In a cohort described by Clissold et al., 8 out of 20 patients with the 17q12 microdeletion (40%) exhibited NDD-related clinical manifestations, whereas none of the individuals carrying intragenic *HNF1B* mutations showed neuropsychiatric alterations, thereby reinforcing the hypothesis of a specific association between 17q12 deletion and neurological vulnerability [25].

Despite the consistent association, the clinical penetrance of the 17q12 deletion appears to be highly variable. Marked phenotypic heterogeneity has been observed even among family members carrying identical chromosomal deletions, including cases of epilepsy and intellectual disability in offspring and mild or absent neurocognitive symptoms in carrier parents. This variability supports the involvement of genetic or environmental modifiers influencing phenotypic expression.

Reported clinical features in affected individuals include global developmental delay, intellectual disability (IQ < 70), learning disorders, expressive language impairment, attention deficit/hyperactivity disorder (ADHD), and autistic traits, with some individuals meeting full diagnostic criteria for autism spectrum disorder (ASD). The 17q12 deletion also confers a high risk of psychiatric disorders, such as schizophrenia and bipolar disorder. EEG monitoring must be carried out because about 10% of the children develop epilepsy [30,31,32].

## 7. Gastrointestinal Features

*HNF1B* is a gene highly expressed in the progenitors of the pancreatic cells during embryonic life and it regulates acinar cell identity and duct morphogenesis [33]. For this reason, it is not surprising that *HNF1B* loss-of-function often leads to structural anomalies of the pancreas, such as pancreas hypoplasia and absence/hypoplasia of the pancreatic body and tail. Biochemical exams can display fecal elastase deficiency, vitamin D deficiency, and vitamin E deficiency [20,34].

As its extended name indicates, *HNF1B* functions as a transcriptional activator in liver. Its haploinsufficiency is associated with liver dysgenesis in about 50% of the patients, and reduced development of the intrahepatic bile ducts and the presence of bile duct cysts are the most frequent defects [35]. Patients with both *HNF1B* SNVs and 17q12 deletions have been reported with liver abnormalities, ranging from an asymptomatic elevation of hepatic transaminase enzyme levels to severe cholestasis. The milder phenotype, with only increased transaminase levels, is more common, but cases of severe cholestasis, also neonatal, have been described. Patients with severe cholestasis can develop end-stage liver disease and need a liver transplant during the pediatric years [36,37].

In a small percentage of cases, gastrointestinal congenital defects occur. Duodenal atresia and short esophagus have been reported [38,39].

## 8. Eye Defects and Hearing Loss

The phenotype of 17q12 deletion includes significant ocular and visual impairments. Ophthalmological manifestations can be observed in up to a third of the cases and are increasingly recognized as an important component of this syndrome. Reported ocular issues in this patient population include coloboma, cataract, strabismus, and optic disc abnormalities such as optic nerve hypoplasia or atrophy, which can lead to reduced visual acuity and visual field defects. Furthermore, retinal dystrophy has been observed in some individuals, suggesting a broader impact on ocular development and function. Anterior segment dysgenesis, including iris abnormalities and glaucoma, has also been documented, highlighting the diverse range of ocular structures that can be affected. [39,40].

Patients occasionally present with sensorineural hearing loss, usually mild to moderate. Both ocular and audiological features are mainly associated with the chromosomal deletion, rather than with *HNF1B* variants.

## 9. Other Congenital Defects

*HNF1B* loss-of-function is a relatively common cause of congenital uterine abnormalities. A study based on 108 probands with uterine congenital defects showed that 8% of them carried a *HNF1B* variant or a deletion encompassing *HNF1B*. For this reason, we can assume that in patients with 17q12 deletions, the haploinsufficiency of *HNF1B* is the primary cause of these malformations [41]. This hypothesis has been confirmed by experiments in mice with loss of *HNF1B*, which resulted in hypoplastic development of the uterus, as well as kidney anomalies [42]. Mayer–Rokitansky–Küster–Hauser syndrome, characterized by aplasia or hypoplasia of the uterus and vagina, is the most common genital defect associated with MODY5, but bicornuate uterus and uterus didelphys are also reported [43,44].

In up to 25% of male patients, cryptorchidism, hypospadias and minor genital defects are described. In this case, it is worth mentioning that genital anomalies mainly occur in males with 17q12 deletions rather than in males with *HNF1B* single nucleotide variants [11,20,45].

Congenital heart defects are not frequent features of MODY5, but, collectively, are reported in more than 10% of the patients. There is no specific heart malformation representing a pathognomonic sign, but the aortic valve and aortic root are more frequently implied. The patients can present with aortic stenosis, increased aortic root size, aortic insufficiency, and septal defects [20,46,47].

## 10. Prenatal Counseling

Prenatal diagnosis of Renal Cysts and Diabetes (RCAD) syndrome can be made in two different scenarios: the diagnosis can be incidental, when a chromosomal microarray analysis (CMA) on amniocytes or chorionic villus is performed for any reason and the fetus is affected by a 17q12 deletion, or the diagnosis can be made after the observation of clinical features evocative of RCAD.

When an affected fetus shows no clinical signs prenatally and the molecular defect is a point mutation of *HNF1B*, the probability of a prenatal diagnosis is very low. Congenital abnormalities of the kidney and urinary tract are the most common features that can be observed prenatally. Bilateral hyperechogenic kidneys and renal cysts are more frequently observed [47,48]. Nonspecific dysplasia, enlarged kidneys, hydronephrosis, pelvic kidney with hydroureter, and lower urinary tract obstruction have also been reported. Kidney abnormalities can be associated with oligohydramnios or anhydramnios. Occasionally, congenital heart defects can be recognized, including transposition of the great arteries, pulmonary valve defect and aortic abnormalities, but these features are very non-specific [47,49].

A prenatal detection of kidney cysts should always induce the clinician to perform genetic tests on fetal DNA, including the CMA, and the study of a panel of genes, including *HNF1B*, involved in the polycystic kidney diseases. A prompt genetic diagnosis is important to provide the parents with information useful to decide about the continuation of the pregnancy.

A genetic diagnosis also can help to plan a dedicated follow-up for the management of the pregnancy, considering that there is a higher risk of premature birth and low birth weight, consistent with reduced insulin secretion in utero. Moreover, an appropriate schedule of screening is necessary for the baby in the first months of life, including screening for congenital defects, the evaluation of psychomotor development, and glucose monitoring, as cases of neonatal hyperglycemia are reported [50].

Most *HNF1B* mutations and 17q12 deletions arise de novo. However, in a significant proportion of cases, RCAD is inherited from a mildly affected parent. For this reason, the genetic tests should always be extended to the parents of the proband. As for all autosomal dominant conditions, an affected individual has a 50% risk of passing it to offspring. On the other side, the risk for parents of a patient with a de novo mutation/deletion is low since it may be related to rare events of gonadal mosaicism.

## 11. Postnatal Counseling

In cases where the renal phenotype does not show up prenatally, the diagnosis usually occurs in children with neurodevelopmental disorders. Chromosomal microarray analysis is usually offered as the first diagnostic test in children with developmental delay, autism and other neurobehavioral signs that can be associated with 17q12 deletions. The diagnosis in patients with *HNF1B* single-nucleotide-variants can be achieved at a later age because neurodevelopmental features are usually absent in these patients. Kidney cysts or echogenic kidneys become evident after childhood in about one third of the patients, whereas diabetes can be diagnosed only in the young adult. Whole exome sequencing or specific gene panels are the most commonly used diagnostic tests.

After the diagnosis, the counselor should stress the point that both RCAD and 17q12 deletion syndrome are characterized by a significant phenotypic variability, even within a family. The range, severity and age of onset of the clinical features cannot be exhaustively predicted after the detection of the genetic defect. Given this premise, the identification of the molecular defect underlying the phenotype is mandatory for more accurate counseling of the parents of the patient. The outcome of patients with *HNF1B* SNVs is usually more favorable, because the intellectual development is usually normal and the rate of congenital defects is lower, as previously described.

A multidisciplinary follow-up must be provided, including:

1. Extended evaluation of both renal morphology and function, including the diagnosis of hypomagnesemia
2. Endocrinologic follow-up for MODY5
3. Neurodevelopmental assessment in children with 17q12 deletions
4. Ultrasound screening for heart and genital defects
5. Eye examination and audiological evaluation
6. Liver function tests
7. Genetic counseling for follow-up care coordination, the identification of eventual less common clinical signs, and providing information about recurrence risk.

For many of the clinical features discussed above, including neurodevelopmental disorders, eye defects and hearing loss, standard treatments are performed. As previously discussed, diabetes can initially respond to oral antihyperglycemic drugs, but insulin is needed over time in most of the cases [51]. Heart and genital malformations may need surgical treatment. Concerning the renal phenotype, a high variability is observed, as previously discussed; the residual function of the kidneys is sufficient for many patients, while others evolve to end-stage renal disease, necessitating dialysis or kidney transplantation. Liver transplantation is very rare, but it has been necessary in the few patients with severe cholestasis and liver insufficiency. After a transplant surgery, a more strict surveillance of plasma glucose is needed: immunosuppressive therapy can trigger diabetes in nondiabetic patients, or worsen the hyperglycemia in diabetic patients, because steroids decrease insulin sensitivity and other immunosuppressive drugs inhibit insulin transcription and/or secretion [35].

The future prospects and ongoing research on the treatment of patients with MODY5 or with 17q12 deletions encompassing the *HNF1B* gene are multifaceted, focusing on precision medicine approaches and a deeper understanding of the underlying pathophysiology. One significant area of investigation involves the advancements in glycemic control strategies. Although specific therapies directly targeting the pancreatic β-cell dysfunction associated with *HNF1B* loss-of-function are still in the early stages of development, some innovative treatments have been proposed [52]. Similarly, for renal manifestations, ongoing nephroprotective strategies and potentially regenerative medicine approaches for kidney disease are being explored [8]. Gene-editing technologies, while still largely experimental for a lot of monogenic disorders, represent a long-term future prospect for correcting the underlying genetic defect.

## Figures and Tables

**Table 1:** This table summarizes all the *HNF1B* variants reported to the HGMD database. The variants are indicated as: missense (M), nonsense and frameshift (N), splicing variants (S), synonymous (Sy), small indels (I), deletion of one or more codons (D).

	Variant	Type		Variant	Type		Variant	Type		Variant	Type		Variant	Type
1	c.3G>T	N	2	c.18delG	N	3	c.22delC	N	4	c.25C > T	N	5	c.30_33delAGAA	N
6	c.34C>T	M	7	c.36_38delCCT	D	8	c.46delC	N	9	c.58G>A	M	10	c.61dupG	N
11	c.70delG	N	12	c.73G>T	M	13	c.79G>C	M	14	c.107C>T	M	15	c.110dupC	N
16	c.118G>A	M	17	c.121_122insAG	N	18	c.130G>A	M	19	c.130G>T	N	20	c.143delT	N
21	c.148C>T	M	22	c.179C>G	M	23	c.182T>G	M	24	c.187delC	N	25	c.188_191 dupATAC	N
26	c.206_207delAC	N	27	c.207_211 delCGCCA	N	28	c.211_217 delAAGGGCC	N	29	c.226G>T	M	30	c.231C>G	M
31	c.232G>T	M	32	c.234G>C	N	33	c.239C>G	M	34	c.241G>T	N	35	c.244G>A	M
36	c.248G>A	M	37	c.252delC	N	38	c.263C>T	M	39	c.264_265delAC	N	40	c.274C>T	M
41	c.280delG	N	42	c.281_284 dupAGCT	N	43	c.286C>T	N	44	c.301G>T	N	45	c.305A>G	M
46	c.313G>A	M	47	c.322delG	N	48	c.324_340del17	N	49	c.329T>G	M	50	c.335G>C	M
51	c.335_342 delGGATGCTC	N	52	c.344+2_344+5 delTAGG	S	53	c.344+2T>C	S	54	c.344+2T>G	S	55	c.345-4C>T	S
56	c.345-1G>A	S	57	c.345-1G>T	S	58	c.353delC	N	59	c.354T>G	Sy	60	c.356G>A	N
61	c.364G>T	M	62	c.367A>T	N	63	c.372G>T	M	64	c.374T>C	M	65	c.377A>G	M
66	c.386_392 delTGCAGCA	N	67	c.388C>T	N	68	c.390_395 delGCAACA	D	69	c.391C>T	N	70	c.393A>T	M
71	c.395A>G	M	72	c.395A>C	M	73	c.398A>G	M	74	c.406C>G	M	75	c.406C>T	N
76	c.412G>A	M	77	c.434T>A	M	78	c.434delT	N	79	c.436A>G	M	80	c.439C>G	M
81	c.439C>T	N	82	c.441G>T	M	83	c.443C>T	M	84	c.443C>G	M	85	c.451T>C	M
86	c.452C>G	M	87	c.454delC	N	88	c.457C>A	M	89	c.460C>T	M	90	c.466A>G	M
91	c.471delC	N	92	c.472_473 insTGCAGCCC	N	93	c.473C>A	M	94	c.475C>G	M	95	c.476C>T	M
96	c.477delT	N	97	c.478A>G	M	98	c.481A>T	N	99	c.484delA	N	100	c.487delC	N
101	c.490A>C	M	102	c.493C>T	M	103	c.493C>G	M	104	c.494G>A	M	105	c.494G>C	M
106	c.495_496delTG	N	107	c.499G>A	M	108	c.499_504 delGCTCTG insCCCCT	I	109	c.503T>C	M	110	c.505T>C	M
111	c.508A>C	M	112	c.512G>A	N	113	c.513G>C	M	114	c.513G>A	N	115	c.516C>G	N
116	c.517G>A	M	117	c.517G>C	M	118	c.523A>T	N	119	c.526C>T	N	120	c.529C>T	N
121	c.530G>A	M	122	c.534delG	N	123	c.541C>T	N	124	c.542G>A	M	125	c.542_544+16 del19	D
126	c.544C>T	N	127	c.544+1G>A	S	128	c.544+1G>C	S	129	c.544+1G>T	S	130	c.544+2dupT	S
131	c.544_544+3 delCGTA	S	132	c.544+3_544+6 delAAGT	S	133	c.544+4A>C	S	134	c.578T>C	M	135	c.589A>C	M
136	c.656C>T	M	137	c.662A>T	M	138	c.674A>C	M	139	c.684C>G	M	140	c.694C>T	M
141	c.695delG	N	142	c.698G>A	M	143	c.703C>T	M	144	c.704G>A	M	145	c.708C>T	Sy
146	c.712T>C	M	147	c.713G>T	M	148	c.715G>C	M	149	c.715_717 delGGG	D	150	c.716G>A	M
151	c.716G>T	M	152	c.717dupG	N	153	c.717delG	N	154	c.719_720dupCC	N	155	c.721G>A	M
156	c.722C>T	M	157	c.727delC	N	158	c.728A>C	M	159	c.731A>G	M	160	c.737T>C	M
161	c.742C>G	M	162	c.742C>T	N	163	c.750C>A	N	164	c.755G>A	M	165	c.758A>C	M
166	c.766C>T	M	167	c.780G>C	M	168	c.781A>G	M	169	c.786_787dupGG	N	170	c.791T>C	M
171	c.791dupT	N	172	c.793G>A	M	173	c.799G>C	M	174	c.809+1G>A	S	175	c.810-4C>G	S
176	c.810-2A>C	S	177	c.810-1G>A	S	178	c.810-1G>C	S	179	c.818G>A	M	180	c.823C>T	N
181	c.826C>G	M	182	c.826C>T	N	183	c.827G>A	M	184	c.840dupC	N	185	c.840delC	N
186	c.840_844 delCTCCA	N	187	c.850delC	N	188	c.853G>A	M	189	c.854G>A	M	190	c.856C>G	M
191	c.857T>G	M	192	c.857T>C	M	193	c.860G>T	M	194	c.865A>G	M	195	c.865A>C	M
196	c.865_870 delAACTTGinsGT	I	197	c.867C>G	M	198	c.869T>A	N	199	c.882_888 delCCGTGTC	N	200	c.883C>T	M
201	c.884G>A	M	202	c.884G>C	M	203	c.886G>T	M	204	c.895T>G	M	205	c.904A>G	M
206	c.905delA	N	207	c.906C>A	M	208	c.908G>A	M	209	c.910A>G	M	210	c.913A>G	M
211	c.913A>T	N	212	c.928C>T	M	213	c.931C>T	N	214	c.940G>A	M	215	c.944T>C	M
216	c.949G>A	M	217	c.949delG	N	218	c.953dupA	N	219	c.967dupA	N	220	c.972_973delCA	N
221	c.981C>G	M	222	c.982_986 delCCTCT	N	223	c.983delC	N	224	c.988C>T	M	225	c.1000T>A	M
226	c.1006delC	N	227	c.1006dupC	N	228	c.1006C>A	M	229	c.1006C>G	M	230	c.1006C>T	M
231	c.1007A>G	M	232	c.1024T>C	M	233	c.1025C>T	M	234	c.1027C>T	M	235	c.1033A>G	M
236	c.1045+1G>A	S	237	c.1045+12T>C	S	238	c.1046-15T>A	S	239	c.1046-2A>G	S	240	c.1046delG	N
241	c.1048dupG	N	242	c.1052_1059 delGCTACAGC	N	243	c.1054T>A	M	244	c.1055A>G	M	245	c.1055dupA	N
246	c.1060C>T	N	247	c.1084T>C	M	248	c.1085C>T	M	249	c.1099A>G	M	250	c.1101T>C	Sy
251	c.1103_1116del14	N	252	c.1107+1G>T	S	253	c.1107+2T>A	S	254	c.1107+2T>C	S	255	c.1108-42G>T	S
256	c.1108-2A>G	S	257	c.1108-2A>T	S	258	c.1108G>T	S	259	c.1108G>A	M	260	c.1117G>C	M
261	c.1127C>T	M	262	c.1128_1129 insCCCCC	N	263	c.1130G>T	M	264	c.1132dupC	N	265	c.1133A>C	M
266	c.1136C>T	M	267	c.1136C>A	N	268	c.1138delG	N	269	c.1144C>T	N	270	c.1147C>T	N
271	c.1156C>T	M	272	c.1196delA	N	273	c.1206+1G>C	S	274	c.1206+5G>C	S	275	c.1207-1G>C	S
276	c.1211_1212delCA	N	277	c.1217C>T	M	278	c.1235delC	N	279	c.1235dupC	N	280	c.1253A>T	M
281	c.1253A>C	M	282	c.1282C>T	N	283	c.1299delC	N	284	c.1302delC	N	285	c.1309+48C>T	S
286	c.1309+54C>T	S	287	c.1310-2A>G	S	288	c.1310-1G>A	S	289	c.1310C>T	M	290	c.1312dupC	N
291	c.1325T>C	M	292	c.1333_1334delGC	N	293	c.1339+1G>A	S	294	c.1339+2dupT	S	295	c.1339+3_1339+6 delAAGT	S
296	c.1339+5G>A	S	297	c.1339+5G>C	S	298	c.1340-276T>C	S	299	c.1340-3C>G	S	300	c.1340-1G>A	S
301	c.1351T>A	M	302	c.1360C>T	N	303	c.1360_1361 delCA	N	304	c.1363_1364 delAG	N	305	c.1367T>C	M
306	c.1370C>T	M	307	c.1373T>G	M	308	c.1390G>C	M	309	c.1395C>G	M	310	c.1397_1412del16	N
311	c.1401delA	N	312	c.1406_1413 dupTGCAGCCC	N	313	c.1408C>T	N	314	c.1414G>A	M	315	c.1446_1459del14	N
316	c.1447C>T	N	317	c.1460T>C	M	318	c.1462C>T	N	319	c.1474G>A	M	320	c.1484T>A	M
321	c.1489delC	N	322	c.1492C>T	N	323	c.1501A>G	M	324	c.1501+1G>A	S	325	c.1501+1G>T	S
326	c.1501+4A>G	S	327	c.1501+5G>C	S	328	c.1501+7G>A	S	329	c.1502-6G>A	S	330	c.1502-2A>G	S
331	c.1502-2A>T	S	332	c.1535-48C>T	S	333	c.1535-23C>T	S	334	c.1538A>G	M	335	c.1540G>A	M
336	c.1543C>T	M	337	c.1547delA	N	338	c.1563G>C	M	339	c.1580G>A	M	340	c.1594A>G	M
341	c.1610C>A	M	342	c.1623+1G>A	S	343	c.1623+2T>C	S	344	c.1624-1G>A	S	345	c.1640C>T	M
346	c.1654-2A>T	S	347	c.1657C>A	M	348	c.1768+1G>A	S	349	c.1768+1G>T	S	350	c.1768 + 3G>A	S
351	c.1769-3C>T	S	352	c.1769-1G>A	S									
